# New ESC/EACTS guidelines for the management of aortic valve disease: key takeaways and implications of long-term TAVR results based on recent explant and redo-TAVR registry outcomes

**DOI:** 10.1186/s13019-026-04643-3

**Published:** 2026-08-06

**Authors:** Saptarshi Paul

**Affiliations:** https://ror.org/00nh9x179 grid.416353.6 0000 0000 9244 0345 St. Bartholomew’s Hospital, West Smithfield, London, EC1A 7BE UK

**Keywords:** Aortic regurgitation, Aortic stenosis, ESC/ EACTS, TAVR, TAVR explant, Redo TAVR

## Abstract

**Background:**

The 2025 European Society of Cardiology (ESC) and European Association for Cardio-Thoracic Surgery (EACTS) guidelines for valvular heart disease represent a pivotal update, emphasizing earlier intervention for aortic stenosis (AS) and broader indications for transcatheter aortic valve replacement (TAVR). These changes are driven by randomized trial data demonstrating TAVR’s safety in lower-risk patients. However, as TAVR expands to younger cohorts, long-term durability remains a concern.

**Main body:**

This narrative review examines key guideline updates for aortic valve disease, focusing on aortic stenosis (AS) and aortic regurgitation (AR), and integrates insights from recent registries (2020–2025) on TAVR explantation and redo-TAVR outcomes. Data from the EXPLANT-TAVR, EXPLANTORREDO-TAVR and FRANCE 2/FRANCE TAVI registries reveal reintervention rates of 0.3–1.7% at 8 years, with redo-TAVR offering superior short-term survival (30-day mortality 1.5–6.2%) compared to explantation (11.9–13.6%). Structural valve deterioration (SVD), leaflet thrombosis, and endocarditis predominate failure causes, underscoring the need for lifetime management strategies.

**Conclusion:**

Implications include refined patient selection, enhanced imaging surveillance, and Heart Team-driven decisions to balance procedural benefits against reintervention risks.

## Background

Aortic valve disease, predominantly AS and AR, affects millions globally, with AS alone impacting approximately 9 million individuals [1] and projected to rise with aging populations. Transcatheter aortic valve replacement (TAVR) has revolutionized management, expanding from high- to low-risk patients following landmark trials like PARTNER 3 [2], Evolut Low Risk [3] and more recently, NOTION-2 [4] and DEDICATE-DZHK6 [5].

The 2025 ESC/EACTS guidelines [6], published August 29, 2025, in the *European Heart Journal*, refine recommendations based on mid-term TAVR data, lowering age thresholds for TAVR in low-risk AS patients and endorsing proactive intervention in asymptomatic severe AS, either by TAVR or SAVR.

## Main text

In spite of the growing usage of TAVR and revision of guidelines, durability concerns of TAVR valves do persist. Bioprosthetic valve failure (BVF) after TAVR, including structural valve deterioration (SVD), thrombosis, and paravalvular leak (PVL), necessitates reintervention in 0.3–1.7% of cases within 8 years. Recent registries (2020–2025) from EXPLANT-TAVR [7], EXPLANTORREDO-TAVR [8], FRANCE 2/FRANCE TAVI [9] provide critical real-world data on explantation and redo-TAVR outcomes, outlining implications of ever evolving guidelines. This review distills guideline takeaways for aortic valve management and evaluates long-term TAVR results, emphasizing clinical implications for Heart Teams.

### Methods

The manuscript has been written in a narrative review format with the aim to determine the judicious application of management techniques for AS during one’s lifetime, taking into account that an individual needs the minimum number of interventions with an acceptable safety profile. A literature search was done for recent (2020–2025) large multi-centre TAVR explant registries, registries providing data about long term TAVR survival and studies providing real world data about redo-TAVR vs. TAVR explant outcomes. The data was manually checked and juxtaposed against the recommendations of the 2025 ESC/ EACTS guidelines.

### Key takeaways from the 2025 ESC/EACTS guidelines for aortic valve disease

The 2025 guidelines [6] prioritize multidisciplinary Heart Team evaluation in specialized Valve Centres (≥ 100 annual aortic interventions), advanced imaging (3D echocardiography, cardiac CT, MRI), and patient-centered lifetime planning, incorporating life expectancy and sex-specific risks (e.g., underdiagnosis in women with low-flow AS). Updates reflect trials like DEDICATE-DZHK6 [5] and NOTION-2 [4], confirming TAVR’s mid-term efficacy in low-risk patients (Table 1).

**Table 1:** Summary of TAVR explant registries with durability evidence

Registry	Period	N (Reinterventions)	Key findings: incidence, modes, outcomes
EXPLANT-TAVR [7]	2009–2020	269 (explant)	Incidence of reintervention: 0.3–0.4%; Endocarditis (43%), SVD (20.1%), PVL (18.2%), PPM (10.8%); 30-day mortality 13.1%; 1-year 28.5%; Redo-TAVR infeasible in 26.8% (unfavourable anatomy).
EXPLANTORREDO-TAVR [8]	2009–2022	396 (181 explant, 215 redo)	Incidence: 0.59%; SVD reason more for redo (63.7% vs 51.9%); PPM reason more for explant (17.1% vs 0.5%); Redo: 30-day mortality 3.4%, 1-year 15.4%; Explant: 13.6% and 32.4% (P<0.001); Similar long-term mortality in both groups post-30 days.
FRANCE 2/FRANCE TAVI [9]	2010–2022	72,850 (793 reinterventions; 591 redo, 111 explant), low rates of Bentall intervention	8-year incidence 1.7%; Early reintervention (62.1%) if post-TAVR gradient >20 mmHg; Explant 6-year mortality 76.2% vs redo 64.0%, P 0.77

### Aortic stenosis (AS)

Severe AS remains the most common indication for intervention, with proactive timing to prevent left ventricular (LV) decompensation.

- **Symptomatic severe AS (Low Surgical Risk)**: TAVR is now Class I (LoE A) for patients ≥ 70 years with tricuspid anatomy and suitable access, down from ≥ 75 years in 2021; surgical AVR (SAVR) is preferred < 70 years (Class I, LoE B). Heart Team decisions integrate frailty, anatomy, and durability.
- **Asymptomatic severe AS (Low Procedural Risk)**: As per the 2025 guidelines, intervention in asymptomatic patients with severe AS and low procedural risk when the LVEF is ≥ 50%, should be considered when a few specific high risk parameters are present (Class IIa, LoE B). This has been a new, broader indication compared to the 2021 guidelines, which advocated consideration of intervention if the high risk features were present in the setting of LVEF > 55% and normal exercise test (Class IIa, LoE B).
- **Bicuspid AS**: TAVR has been downgraded to Class IIb (LoE B) for increased surgical risk with suitable anatomy, due to limited durability data; SAVR remains first-line for younger patients.
- **Coronary assessment**: For CAD assessment before valve intervention, CCTA is recommended (Class I, LoE B) in patients with moderate/low (≤ 50%) pre-test likelihood to rule out obstructive CAD, whereas invasive coronary angiography is recommended (Class I, LoE C) in patients with high/very high (> 50%) pre-test likelihood. In the 2021 guidelines, CCTA was supposed to be considered as an alternative to conventional angiography for valve surgery in patients with severe valvular heart disease with low probability of CAD (Class IIa, LoE C).

Antithrombotic therapy post-TAVR avoids dual antiplatelet therapy (DAPT) or direct oral anticoagulants (DOACs) routinely (Class III), unless indicated (e.g., atrial fibrillation).

### Aortic regurgitation (AR)

- **Severe symptomatic AR (Inoperable)**: TAVR is a new Class IIb (LoE B) option for selected inoperable AR patients at experienced centers using dedicated devices only. This is a new inclusion based on feasibility studies.
- Asymptomatic thresholds for surgery in case of severe AR have incorporated a slightly different approach from the 2021 guidelines, with a Class IIa indication for surgery if the LVEF ≤ 50%, with an LVESDi > 25 mm/m^2^, and a Class IIb indication for surgery based on an LVEF ≤ 55% and an LVESDi > 22 mm/m^2^ and LVESVi > 45 ml/m^2^ (especially with a BSA < 1.68 m^2^).

These updates promote equity, reducing under-treatment via earlier referrals and education on anticoagulation adherence (Class I, LoE A).

### Long-term TAVR outcomes: insights from recent explant and redo-TAVR registries (2020–2025)

As TAVR volumes exceed 1 million annually, incidence rates of bioprosthetic valve failure (BVF) from the redo-TAVR and TAVR explant registries have been under strict scrutiny. Cumulative reintervention incidence is low (0.3–1.7% at 8 years) but rising with younger patients. Key indications for reintervention include SVD (52–64%; leaflet calcification/tear), PVL (19–33%), endocarditis (43% in explants), and prosthesis-patient mismatch (PPM; 11–17%). Subclinical leaflet thrombosis (hypoattenuated leaflet thickening [HALT]) has been rated at 9–24% within 1 year, potentially accelerating calcification, though its prognostic impact varies (no increased mortality in some cohorts up to 5 years) [7, 9–11].

Redo-TAVR (valve-in-valve) yields high success (79–85%), low embolization (2–3%), and stroke (1–2%), with median time to reintervention 46 months (vs. 18 for explant). Balloon vs. self-expanding valves show comparable durability, but bicuspid anatomy limits redo feasibility. Explantation, often requiring root surgery (13%), carries higher perioperative risks, particularly in endocarditis. Mortality post explant is high, often bordering prohibitive, as compared to redo-TAVR [8, 9, 12].

## Discussion

The 2025 guidelines’ endorsement of TAVR ≥ 70 years aligns with registry data showing low reintervention rates (0.3–1.7%). However, these rates are heavily influenced by competing mortality, survivor bias, heterogeneous devices/eras, and anatomic feasibility for redo-TAVR. For patients < 70 years or with bicuspid valves, SAVR’s durability edge (lower SVD risk) favours initial surgery, as redo-TAVR feasibility drops (anatomic mismatch in 27–40%) [6–9].

### How to develop a lifetime model?

The pertinent question, hence, is to develop a robust lifetime model for primary intervention and reintervention in patients, keeping in mind that the total number of interventions should be at a minimum. Overall, SAVR remains superior compared to TAVR because of redo feasibility [13] and durability issues affect those who have already received TAVR or receiving TAVR as a part of a low-surgical-risk cohort. We have inevitably nursed a large burden of patients who have longer life expectancies but failing valves, thus exposing them to interventions with poor long term results, thus transpiring to shorter survival. Redo TAVR, although carries a more favourable risk profile, is unsuitable in many cases because of hostile anatomy, forcing a steer towards TAVR explants. As Lee, et al. have pointed out, TAVR explant is the fastest growing surgery in the United States currently [12], and Bowdish, et al. have noted a dramatic rise (> 4000%) in cardiac surgeries post TAVR [14]. The graph shows a steep rise post guideline amendments in 2020 when TAVR was first labelled a Class I recommendation based on an age/life-expectancy framework with shared decision-making in the “Intermediate zone” of 65–80 year range [14, 15]. Braasch, et al., however, have noted that redo-TAVR constitutes a substantial proportion of late reinterventions (beyond 5 years from the index TAVR) as compared to TAVR explants [16].

Data may lead to believe that redo-TAVR’s safety outperforms explantation, with similar 1-year survival rates. However, its apparent safety advantage is largely confined to early outcomes, with longer-term mortality potentially favouring surgical approaches. Again, patient selection in redo TAVR gets largely limited by prohibitive coronary anatomy, multivalvular involvement, severe patient-prosthetic mismatch, or endocarditis, when TAVR explant remains as the only option. Lee, et al., have stated the 30-day mortality rates of TAVR explant of upto 20%, and 1-year mortality rates ranging from 20% to 30% [12].

In AR, emerging TAVR feasibility (Class IIb) must weigh a higher thrombosis risk. Latest guidelines state an eligibility for TAVR for strictly symptomatic AR patients who are in the high/ prohibitive surgical group. Because of limited durability data, guidelines are understandably conservative with no scope for TAVR intervention in asymptomatic patients reaching LV thresholds for intervention [6]. Off label devices like Medtronic Evolut Pro or Edwards Sapien 3 (designated for AS) rely entirely on oversizing and radial force to wedge the valve in a dilated, calcium-free annulus [17], hence often struggle with higher rates of device migration and paravalvular leaks [18], unlike the on-label devices like the J-valve or the Jena valve [18].

### Future directions

As we look into the future, trials like EARLY TAVR for asymptomatic AS will give us more insight regarding the scope of TAVR in asymptomatic severe AS patients [19]. Recent publications have reviewed next generation devices (Siegel THV and Optimum THV) wherein the early feasibility trials show < 1% incidence of SVD in 1 year [20]. Also, in the COMMENCE trial of surgical Inspiris Resilia valves (*n* = 689), Resilia tissue demonstrated a marked reduction in structural valve deterioration-related hemodynamic valve deterioration (HVD) at five years, with grade ≥ 2 HVD occurring in only 1.8% of the patients versus 3.5% in a contemporary surgical control arm (one-sided 95% lower-bound hazard ratio 1.15; *p* = 0.03) [21].

## Conclusion

The 2025 ESC/EACTS guidelines advance aortic valve management by promoting TAVR’s role in earlier, less invasive care, supported by favourable redo-TAVR outcomes from recent registries. However, durability data underscore the imperative for lifetime strategies, vigilant imaging, and selective SAVR in young/bicuspid patients to minimize high-risk explants. Heart Teams must educate on reintervention realities to optimize intervention and outcomes. Individual interventions must be patient specific rather than incorporating a blanket intervention.

## Data Availability

No datasets were generated or analysed during the current study.

## Abbreviations

TAVR: Transcatheter aortic valve replacement
SAVR: Surgical aortic valve replacement
ESC: European Society of Cardiology
EACTS: European Association for Cardio-Thoracic Surgery
AS: Aortic stenosis
AR: Aortic regurgitation
PVL: Paravalvular leak
LoE: Coronary computed tomography angiography
CAD: Coronary artery disease
DAPT: Dual antiplatelet therapy
DOAC: Direct oral anticoagulants
BVF: Bioprosthetic valve failure
PVL: Paravalvular leak
LVEF: Left ventricular ejection fraction
LV: Left ventricle
PPM: Prosthesis-patient mismatch
HALT: Hypoattenuated leaflet thickening
THV: Transcatheter heart valve
HVD: Haemodynamic valve deterioration
SVD: Structural valve deterioration
